# Prevalence and associated factors of knee osteoarthritis in a rural Chinese adult population: an epidemiological survey

**DOI:** 10.1186/s12889-016-2782-x

**Published:** 2016-01-30

**Authors:** Yuan Liu, Haifeng Zhang, Ningxia Liang, Weimin Fan, Jun Li, Zuhu Huang, Zhijian Yin, Zhijun Wu, Jun Hu

**Affiliations:** 1 Department of Orthopedics, The First Affiliated Hospital of Nanjing Medical University, Guang Zhou Road 300, Nanjing, 210029 China; 2 Department of Infectious Diseases, The First Affiliated Hospital of Nanjing Medical University, Nanjing, 210029 China; 3 Department of Cardiology, The First Affiliated Hospital of Nanjing Medical University, Nanjing, 210029 China; 4 Centre Clinics of Baqiao, Gaoyou, 225642 China

**Keywords:** Knee osteoarthritis, Epidemiology, Prevalence, Metabolic syndrome, Smoking

## Abstract

**Background:**

The exact pathogenic mechanism of knee osteoarthritis (OA) is still unknown. With the exception of clinical treatment to alleviate symptoms, or total knee replacement, there is currently no effective treatment method. Consequently, an in-depth etiological and epidemiological study of knee OA can provide clues for diagnosis, treatment and scientific research, and will ultimately have a beneficial effect on public health.

**Methods:**

A cross-sectional community study in the rural village of Gaoyou was conducted in 3428 Chinese adults (aged ≥ 40 years). Subjects completed an interviewer-administered questionnaire, evaluating knee pain and associated disability, analgesia, use of health services, past medical history, walking, income, smoking, and use of oral contraceptives, and standardized weight-bearing knee radiographs were obtained. Patient demographic characteristics and biochemical parameters were recorded.

**Results:**

Single-factor regression analysis indicated that age, overweight, central adiposity, high low-density lipoprotein cholesterol (LDLC), high total cholesterol (TC), high triglycerides (TG), dyslipidemia, hypertension and low income were the associated factors for knee OA in females; age, high LDLC, hypertension, low income and frequent walking were the associated factors for knee OA in males. Interestingly, male heavy smokers were less likely to develop severe knee OA compared with non-smokers. Stepwise logistic regression analysis indicated that age and overweight were the associated factors for knee OA for all individuals. Although central adiposity, high LDLC, high TC, high TG, dyslipidemia, hypertension and low income appeared to be related to knee OA in females according to univariate analysis, these factors were not identified in stepwise logistic regression analysis. In addition although age, high LDLC, hypertension and frequent walking were also the associated factors for knee OA in males by stepwise logistic regression analysis, smoking as a protective factor was not identified in this analysis.

**Conclusions:**

In this study, aging, obesity, frequent walking, low income and relevant multiple metabolic disorders were the associated factors for knee OA. Smoking might be associated with a lower prevalence of OA in male smokers according to univariate analysis. A retrospective association of smoking with OA may constitute an important etiologic clue, but further well-designed, large-scale prospective controlled trials are required to confirm these findings.

## Background

Osteoarthritis (OA) is the most common joint disease worldwide, and primarily affects the knees, hips, hands, and spine. It is a leading cause of disability among older individuals aged above 40 years. Besides affecting patients' activity and quality of life, OA will further cause depression and anxiety, as well as a great economic burden [1].

To date, most large population-based epidemiological studies evaluating the disease prevalence of OA have been performed in Europe or North America although it has been estimated that by 2050, almost four fifths of the world’s older population (65 years and older) will be living in less-developed regions of the world [2]. To start to address this disparity, several reports on the prevalence of knee OA in China have been conducted. One study reported that the prevalence of radiographic knee OA (42.8 %) and symptomatic knee OA (15.0 %) in elderly women over 60 years of age in the urban district of Beijing was higher than that in elderly American women of the same age group. In contrast the prevalence of radiographic knee OA and symptomatic knee OA in Chinese males was similar to that in American males [2]. In another study the population over 50 years of age in Wuchuan County of Inner Mongolia was analyzed, and their data were compared with those from Beijing and Framingham (Massachusetts, USA). The results indicated that the prevalence of symptomatic knee OA in the residents of rural Wuchuan County was higher than that in the urban residents of Beijing or Framingham. However most respondents in Wuchuan County were heavy laborers and this county is located in Inner Mongolia, with a unique natural environment. As a result, the prevalence might be unrepresentative [3, 4].

Obesity, hypertension, dyslipidemia, diabetes and insulin resistance tend to cluster into so-called metabolic syndrome (MS). There is growing evidence suggesting that metabolic syndrome (MS) is a risk factor for the development of OA [5–8]. Among the 1334 white patients in a study by Gandhi et al., 114 (8.5 %) had MS as compared with 3 of 36 (8.3 %) blacks and 18 of 90 (20 %) Asians. Adjusted analysis showed that those of Asian ethnicity had double the risk of MS compared with those of other ethnicities. MS is a risk factor for OA, and Asians demonstrate a greater prevalence of MS compared with whites and blacks in this population [9]. However, there are few publications which mention the associated factors, such as metabolic diseases, for knee OA in the Chinese population. Thus, in order to facilitate the epidemiological study of knee OA in China, the rural population of Gaoyou City of Jiangsu Province (located in the economic belt along the Yangtze Delta, 300 km from Shanghai) was sampled in this study.

We performed a cross-sectional study in the Han population above 40 years of age in the rural areas of Gaoyou City to investigate the prevalence of knee OA in males and females and its distribution in each age group. Relevant associated factors, including age, overweight, central adiposity, high low-density lipoprotein cholesterol (LDLC), high total cholesterol (TC), high triglycerides (TG), low high-density lipoprotein (HDL), dyslipidemia, hypertension, diabetes, hyperuricemia, income level, walking habit, smoking habit, and use of oral contraceptives were analyzed. We aimed to provide basic data on the epidemiological features of knee OA in a Chinese population, thus providing a scientific basis for preventing and treating osteoarthritis.

## Patients and methods

Proportionately stratified random sampling was used to select a representative sample from the towns of Baqiao and Songqiao in Gaoyou, a rural area 300 km north of Shanghai, China. The sample was stratified by sex and age based on population data obtained from the local authorities. Ultimately, 5000 subjects in Baqiao and 1000 subjects in Songqiao aged 18 to 75 years old were selected and invited to participate in the study. In the period from January to May 2010, 4536 subjects participated in the present study, including 3918 subjects in Baqiao and 618 subjects in Songqiao; the response rates were 78.4 and 61.8 %, respectively. Of these 4536 respondents, 3428 subjects aged 40–74 years old were selected and invited to participate in the study of prevalence and associated factors of knee osteoarthritis. The protocol was approved by the Ethics Committee of the First Affiliated Hospital with Nanjing Medical University. Informed consent was obtained and signed by each participant [10, 11].

Trained health professionals administered the survey questionnaires because it was anticipated that many study participants would be illiterate. All interviewers, clinical examiners, and radiography technicians were trained under the supervision of the study’s chief investigators. Participants were interviewed at their homes or site of identification. Trained health professional interviewers administered a standardized questionnaire that focused on joint symptoms, previous diagnoses of arthritis, possible associated factors for OA, and more detailed information on general health status, height, weight, smoking habits, medication, income, use of hormones, specific physical loads from occupation and housework, and sports activities. At the end of the interview, participants were invited to the central examination site for a clinical examination, laboratory testing, and radiography. Transportation to the hospital was provided. All biochemical parameter measures were ascertained at time of study visit using a standard protocol rather than reliance on existing medical and other records.

A weight-bearing posteroanterior view radiograph was taken of both knees strictly according to a validated acquisition protocol [3, 12]. Radiographs were read by the study’s chief investigator using the Osteoarthritis Research Society International atlas, and Kellgren/Lawrence (K/L) grades (range 0–4) were also assigned. We used the same definitions to define cases of symptomatic knee OA as were used in previous studies [2, 3, 13, 14]. Symptomatic knee OA was defined as having at least one knee with both a K/L grade 2 and a positive response to the question, “In the past 12 months, have you had knee pain lasting most days for at least a month?” Knee OA In this study means symptomatic knee OA.

Standing height was measured with a fixed stadiometer calibrated in centimeters. Weight was measured to the nearest 0.1 kg using a balance beam scale with subjects wearing light clothing and no shoes. Body mass index (BMI) was calculated as weight in kilograms divided by height squared in meters. Overweight was defined as BMI ≥ 25 kg/m^2^ in both men and women. Waist circumference was measured (in centimeters) between the lowest rib margin and the iliac crest [15]. Central adiposity was defined as waist circumference ≥ 90 cm in men or ≥ 80 cm in women [16]. A number of laboratory tests, such as LDLC, TC, TG, HDL, fasting plasma glucose, and uric acid were performed. High LDLC was defined as ≥ 4.14 mmol/L. High TC was defined as ≥ 6.12 mmol/L. High TG was defined as ≥ 2.26 mmol/L, and low HDL as ≤ 1.03 mmol/L [17]. Dyslipidemia was diagnosed according to the Guide for Dyslipidemia in China (TC > 5.72 mmol/L and/or TG > 1.70 mmol/L and/or HDL < 0.91 mmol/L) [18].

Blood pressure was measured by trained examiners using a mercury sphygmomanometer according to a standard protocol. After each subject rested for at least 5 min in the sitting position, his or her blood pressure was measured three times on the right arm with the arm cuff maintained at the level of the heart. These three readings were averaged for the analysis. Hypertension was diagnosed if the average of the three blood pressure readings was at least 140 mmHg systolic blood pressure (SBP) or 90 mmHg diastolic blood pressure (DBP), or if the subjects were on antihypertensive medication. Diabetes was diagnosed if the fasting serum glucose concentration was at least 7.0 mmol/L [19] or if the subject was on antidiabetic medication or insulin treatment [20]. Hyperuricemia was defined as uric acid level ≥ 417 μmol/L in men or ≥ 357 μmol/L in women [21].

Low income was defined as income < 500 RMB per month. Frequent walking was defined as walking for 2 miles or more, at least once a week [22]. A semiquantitative measure of light (<10 cigarettes/day), medium (10–20 cigarettes/day) and heavy (>20 cigarettes/day) smoking was performed for a dose–response analysis in the smoking participants. None of the women in the study population smoked, so we did not calculate smoking data for women. The use of oral contraceptives was divided into an exposed group, where the participants had taken pills for one year or more, and an unexposed group, where the participants had taken pills for less than 1 year or not at all.

For database management and statistical analysis, we used SAS software version 9.2 (SAS Institute, Cary, NC, USA). Odds ratios of individual associated factors and their association with OA by sex were calculated by logistic regression analyses. Stepwise logistic regression analyses were used to evaluate the factors that were independently associated with OA in the whole population, and separately in women and men; *P*-values for covariates to be included in the model were set at 0.05. *P*-values < 0.05 were considered statistically significant, and all tests were two-tailed [11].

## Results

**Fig. 1:**
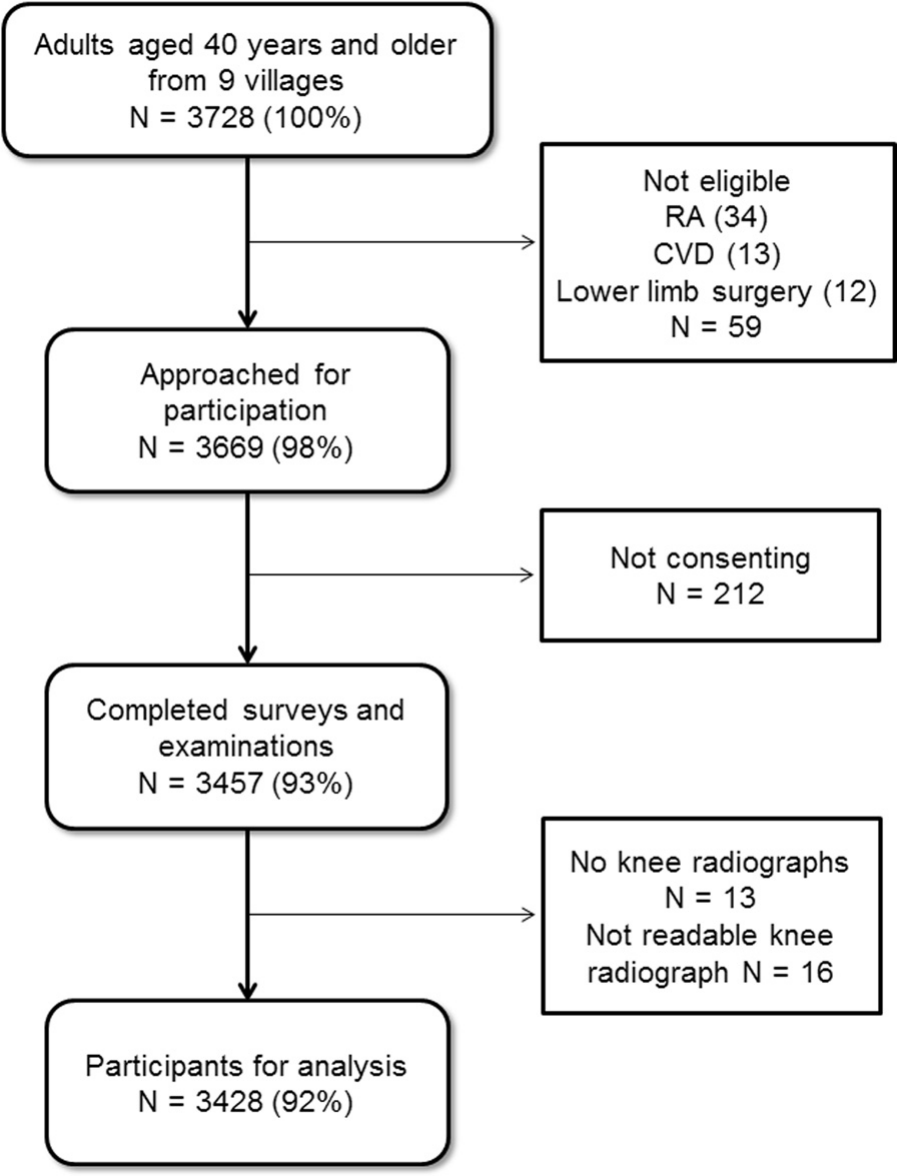
Gaoyou symptomatic knee osteoarthritis study flow chart. RA = Rheumatoid arthritis. CVD = Cerebrovascular disease

In total, 3428 individuals aged 40 years and older were identified in the randomly selected households in Gaoyou County, a rural area 300 km north of Shanghai, China (Fig. 1). Those who declined to participate were older compared with the study participants (mean ± SD age 58 ± 9 years versus 55 ± 10 years). Table 1 show the sex and age distribution of the respondents. There were 568 knee OA cases in the study; 279 in females and 289 in males. The overall prevalence of OA was 16.57 % (15.79 % in women, 17.40 % in men) with no significant sex differences (men: women, odds ratio [OR] 1.12, 95 % confidence interval [CI] 0.93–1.34, *P* = 0.23). The overall prevalence of knee OA increased significantly with age (*P* < 0.01), reaching 29.25 % of women and 24.71 % of men in the age-group 70 years and older. After gender stratification, both male and female knee OA prevalence increased dramatically with age (*P* < 0.01) (Table 1).

**Table 1:** Prevalence of symptomatic knee osteoarthritis stratified by gender and age group in the study population

Age group	Women		Men	
	*n*	Case (%)	*n*	Case (%)
40~	730	70 (9.59)	440	40 (9.09)
50~	516	89 (17.25)	497	96 (19.32)
60~	415	89 (21.45)	554	111 (20.04)
≥70	106	31 (29.25)	170	42 (24.71)
Total	1767	279 (15.79)	1661	289 (17.40)

Table 2 shows the results of univariate analysis of individual associated factors and their association with OA by sex. Among women, the associated factors significantly associated with OA were age, overweight, central adiposity, high LDLC, high TC, high TG, dyslipidemia, hypertension and low income (*P* < 0.05). Among men, the factors showing a significant association with OA were age, high LDLC, hypertension, low income and frequent walking (*P* < 0.05). Interestingly, male heavy smokers were less likely to develop severe knee OA compared with non-smokers (*P* < 0.05). Table 3 shows the results of stepwise logistic regression analysis of associated factors for OA in all the respondents, irrespective of sex. The factors significantly associated with OA in the general population were age and overweight (*P* < 0.05). Table 4 shows the results of stepwise logistic regression analysis of associated factors for OA by sex. The factors significantly associated with OA were again shown to be age in both women and men (*P* < 0.05). However, overweight was only a factor in women, while high LDLC, hypertension, and frequent walking were only significant factors in men (*P* < 0.05).

**Table 2:** Odds ratios (OR) of individual associated factors with symptomatic knee osteoarthritis by sex

Associated factor	Women			Men		
	*n*	Case (%)	OR	*n*	Case (%)	OR
Age						
40~	730	70 (9.59)	1	440	40 (9.09)	1
50~	516	89 (17.25)	1.97 (1.40–2.75)	497	96 (19.32)	2.39 (1.61–3.55)
60~	415	89 (21.45)	2.57 (1.83–3.62)	554	111 (20.04)	2.51 (1.70–3.69)
≥ 70	106	31 (29.25)	3.90 (2.40–6.33)	170	42 (24.71)	3.28 (2.04–5.28)
Overweight						
No	967	134 (13.86)	1	950	163 (17.16)	1
Yes	800	145 (18.13)	1.35 (1.05–1.74)	711	126 (17.72)	1.04 (0.80–1.34)
Central adiposity						
No	1193	116 (9.72)	1	1207	202 (16.74)	1
Yes	574	115 (20.03)	1.53 (1.18–1.99)	454	87 (19.16)	1.18 (0.89–1.56)
High LDLC						
NO	1656	252 (15.22)	1	1558	266 (17.07)	1
Yes	111	27 (24.32)	1.79 (1.14–2.82)	103	23 (22.33)	1.40 (1.06–2.26)
High TC						
NO	1568	239 (15.24)	1	1493	256 (17.15)	1
Yes	199	42 (21.11)	1.44 (1.00–2.08)	168	33 (19.64)	1.18 (0.79–1.77)
High TG						
NO	1518	228 (15.02)	1	1391	247 (17.76)	1
Yes	249	53 (21.29)	1.49 (1.07–2.08)	270	42 (15.56)	0.85 (0.60–1.22)
Low HDL						
NO	1457	236 (16.20)	1	1250	237 (18.96)	1
Yes	310	54 (17.41)	0.84 (0.66–1.32)	411	68 (16.55)	0.65 (0.44–1.25)
Dyslipidemia						
NO	1181	169 (14.31)	1	1024	191 (18.65)	1
Yes	586	111 (18.94)	1.40 (1.08–1.82)	637	98 (15.38)	0.79 (0.61–1.04)
Hypertension						
No	903	122 (13.51)		652	92 (14.11)	
Yes	864	157 (18.17)	1.42 (1.10–1.84)	1009	197 (19.52)	1.48 (1.13–1.93)
Diabetes						
No	1639	257 (15.68)		1539	270 (17.54)	
Yes	128	22 (17.19)	1.12 (0.69–1.80)	122	19 (15.57)	0.87 (0.52–1.44)
Hyperuricemia						
No	1603	251 (15.66)		1412	247 (17.49)	
Yes	164	28 (17.07)	1.11 (0.72–1.70)	249	42 (16.87)	0.96 (0.67–1.37)
Low income						
NO	1571	238 (15.15)		1453	239 (16.45)	
Yes	196	43 (21.94)	1.54 (1.06–2.19)	208	50 (24.04)	1.61 (1.14–2.27)
Often walk						
No	1545	242 (15.66)		1444	241 (16.69)	1
Yes	222	37 (16.67)	1.08 (0.74–1.57)	217	48 (22.12)	1.42 (1.00–2.01)
Smoke						
Nonsmoker				487	92 (18.89)	1
< 10 per/day				241	46 (19.09)	0.91 (0.71–1.34)
10 ~ per/day				405	72 (17.78)	0.82 (0.66–1.21)
20 ~ per/day				528	67 (12.69)	0.62 (0.45–0.86)
Oral Contraceptive Users						
No	1644	263 (16.00)	1	0		
Yes	123	16 (13.01)	0.79 (0.46–1.35)	0		

*LDLC* low-density lipoprotein cholesterol, *TC* total cholesterol, *TG* triglycerides, *HDL* high density lipoprotein

**Table 3:** Stepwise logistic regression analysis of factors associated with symptomatic knee osteoarthritis in the entire study population (both sexes combined)

Variable	OR (95 % CI)	*P* value
Age	1.04 (1.03–1.05)	<0.0001
Overweight	1.05 (1.02–1.08)	0.0024

**Table 4:** Stepwise logistic regression analysis of factors associated with symptomatic knee osteoarthritis by sex

Variable	Women		Men	
	OR (95 % CI)	*P* value	OR (95 % CI)	*P* values
Age	1.05 (1.03–1.06)	<0.0001	1.03 (1.02–1.05)	<0.0001
Overweight	1.05 (1.01–1.09)	0.0228		
High LDLC			1.22 (1.01–1.46)	0.0362
Hypertension			1.01 (1.00–1.01)	0.0211
Often Walk			1.57 (1.10–2.24)	0.0128

*LDLC* low-density lipoprotein cholesterol

## Discussion

Along with the development of the Chinese economy and the associated changes in lifestyle, there is an increase in the aging population and the resulting burden on family and society caused by knee OA has attracted increasing attention. Extensive epidemiological surveys have indicated that the prevalence of knee OA varies greatly with region, race and socioeconomic conditions. Prevalence is also influenced by a variety of environmental and genetic factors. To date, the large-sample epidemiological surveys that have been carried out have mainly concentrated on North America and European regions. There have been few relevant surveys in China, especially in the middle and lower reaches of the Yangtze River region which has a high population density. We carried out an epidemiological survey of knee OA in China to address this gap in knowledge.

The result indicates that age and weight are positively correlated with the prevalence of knee OA, which is in agreement with the results of most other epidemiological surveys [23, 24]. Lohmander et al. [15] carried out a large-sample follow-up survey on discharged patients in Sweden over 10 years. They found that BMI, waist circumference, waist–hip ratio, weight and percentage body fat, which are all closely related to weight, showed a positive correlation with the prevalence of knee OA. Jiang et al. arrived at the conclusion that obesity is a risk factor for knee OA by systematically analyzing the correlation between BMI and knee OA in 21 independent reports [25]. Our result also indicated that the population with high BMI showed a significantly increased prevalence of knee OA, with the increase in waist circumference being closely related to the prevalence of knee OA in females. This may be because the pressure exerted on the articular cartilage increases, which accelerates degeneration. However, some studies have indicated that obesity is also positively correlated with the prevalence of OA in non-load-bearing joints such as in the hand [26]. This means that the load on the joint cannot completely explain the relationship between obesity and knee OA.

Soran et al. [27] found that levels of serum high-density lipoprotein-cholesterol (HDL-C), total thiol (total free sulfhydryl groups, −SH), paraoxonase and arylesterase activities were significantly lower in the OA patient group than in controls, while lipid hydroperoxide (LOOH) and low-density lipoprotein (LDL) levels were significantly higher. Dyslipidemia may result in the ectopic deposition of lipids in particular in chondrocytes, which aggravates lipid metabolism disorders in degenerative articular cells and promotes the development of OA. Thus obesity-related metabolic factors, especially adipokines, can induce the expression and release of inflammatory factors and metabolic enzymes, inhibit the synthesis of articular cartilage and stimulate the remodeling of subchondral bone [5]. Singh et al. found that patients with OA are more likely to have hypertension (40 % vs. 25 %), diabetes (11 % vs. 6 %), high total cholesterol (32 % vs. 24 %), and renal impairment (37 % vs. 27 %) compared with the unaffected population [28]. The mechanism may involve ischemia below the cartilage of knee OA patients caused by hypertension. This type of ischemia can inhibit the metabolism of articular cartilage and trigger bone remodeling.

In the late 1980s, the negative correlation between smoking and OA was shown by a cross-sectional epidemiological study and a cohort study by Felson. Later, Felson et al. continued to investigate these respondents. X-ray images of the knee joint in the load-bearing position were taken in 1983–1985 and again in 1992–1993. The results indicated that the prevalence of OA in the smokers was lower compared with the non-smokers (OR = 0.4, 95 % CI [0.2–0.8]) [13, 14]. In the past 20 years, several research institutes have been observing the relationship between smoking and OA. Samanta et al. [29] found a negative correlation between smoking and large-joint OA according to the data from the Nottingham Clinical Center Database (UK). Jarvholm et al. [24] found a negative correlation between smoking and hip OA in a study of male construction workers in Sweden, but the influence of smoking was weaker than that of BMI and age.

However, some reports suggest that smoking is not obviously related to OA. In 2011, Hui et al. [30] performed a meta-analysis of the existing data concerning smoking and OA. They reported that the results indicative of a negative correlation between smoking and OA were mainly derived from a hospital setting. The protective effect of smoking on the joints needs to be further supported by a normatively designed survey with community-based data. In our survey, data were collected from the community for multi-factor regression analysis, and the results indicate smoking might be associated with a lower prevalence of OA in male smokers according to univariate analysis.

There are several theories concerning the mechanism underlying the protective effect of smoking: 1) nicotine can increase the glycosaminoglycan level and collagen synthesis activity in articular cartilage; 2) nicotine can regulate the anabolic activity of articular cartilage; 3) nicotine can influence the deformability of subchondral bone under impact load by reducing bone density, and therefore reducing the pressure on the cartilage; 4) nicotine can inhibit the inflammatory factors involved in knee OA [31, 32]. It is notable that surveys from several institutes as well as some meta-analyses have indicated that smoking is a risk factor for rheumatoid arthritis, especially for patients with positive rheumatoid factor [33, 34]. Evidently the relevant mechanism between smoking and knee arthritis, such as OA and rheumatoid arthritis, needs to be further explored.

Our study also found that low income and long-term walking increase the prevalence of knee OA. This may be because the low-income population usually engages in heavy physical labor, which increases the load on the knee joints. Similarly, long-term walking also increases loading on the knee joints, thus aggravating any damage to the cartilage [35]. No correlation between oral contraceptive use and knee OA was found in our survey. There have been several studies indicating a possible inverse relationship between estrogen intake and knee OA, but in a study by Sandmark et al., estrogen therapy for women over 50 was associated with an increased relative risk of 1.8 (95 % CI 1.2–2.6), while use of oral contraceptives did not influence the risk [23].

Our study has some limitations. First, radiological examination and symptomatic examination are the common diagnostic methods for the epidemiological investigation of knee OA. In our study, the symptomatic diagnosis method was used as the main inclusion criterion for knee OA, so a questionnaire was the primary screening tool. Inevitably, there were likely to be some differences in the discrimination of information by investigation staff although they had all received the same training. Second, the survey data were epidemiological data from only one small area. More epidemiological data from different areas need to be collected and analyzed in future. Third, the causality between OA and associated factors cannot be confirmed fully by the cross-sectional study method, the results need to be further confirmed by prospective studies.

## Conclusions

In conclusion, aging, obesity, increased knee joint movement, low income and relevant multiple metabolic disorders are important associated factors for OA. Smoking seems to be associated with a lower prevalence of OA, although it has not been included in stepwise logistic regression analysis. Considering the difficulty of epidemiological investigation in rural populations and the availability of study data in this region, we believe the results of our population-based screening study can aid in international comparisons and elucidate the factors associated with knee OA in China.

## Abbreviations

BMI: body mass index
DBP: diastolic blood pressure
HDL: high-density lipoprotein
K/L: Kellgren/Lawrence
LDLC: low-density lipoprotein cholesterol
MS: metabolic syndrome
OA: osteoarthritis
SBP: systolic blood pressure
TC: total cholesterol
TG: triglycerides
